# The nutritional profile comparison between the white and purple Açaí in the mesoregions of Pará, Brazil

**DOI:** 10.3389/fnut.2024.1417076

**Published:** 2024-12-04

**Authors:** Rosemary Maria Pimentel Coutinho, Juliana De Cássia Gomes Rocha, Nathália Neves, Vera Lúcia Dias da Silva, Vitória Nazaré Costa Seixas, Isidro Hermosín-Gutiérrez, Antonio Fernandes de Carvalho, Paulo Cesar Stringheta

**Affiliations:** ^1^Instituto Federal de Educação Ciência e Tecnologia do Pará – Campus Belém, Belém, Pará, Brazil; ^2^Department of Food Technology, Federal University of Viçosa, Viçosa, Minas Gerais, Brazil; ^3^DETA – Department of Food Technology, State University of Pará (UEPA), Belém, Pará, Brazil; ^4^University of Castilla-La Mancha, Ciudad Real, Castilla-La Mancha, Spain

**Keywords:** white açaí fruits, *Euterpe oleracea* Mart., total phenolics, total anthocyanins, antioxidant capacity, mineral composition, microbiological profile

## Abstract

The study targeted to compare the nutritional profile of two varieties of açaí, the white and purple, found in different mesoregions of Pará, Brazil. The research focused on analyzing levels of total phenolics, total anthocyanins, antioxidant capacity, and mineral composition in these two varieties. The study sought to identify significant differences between the two varieties in terms of nutritional composition and antioxidant potential, providing valuable information into the specific nutritional and functional properties of each type of açaí studied. Higher levels of total phenolics, total anthocyanins, and antioxidant capacity were observed in purple açaí fruits, with values of 806.17 ± 17.48 mgGAE/100 g, 81.73 ± 1.77 mg/100 g, and 19.25 ± 0.35 μmol of Trolox equivalent (TE)/g, respectively, compared to 401.92 ± 52.70 mgGAE/100 g, 37.70 ± 5.34 mg/100 g, and 6.17 ± 1.07 μmol TE/g in white açaí. HPLC-MS analysis identified and quantified monomeric anthocyanins in white açaí, using two distinct analytical methods, revealing average values of 0.29 and 1.05 μg/100 g for cyanidin-3-glucoside and between 0.74 and 3.13 μg/100 g for cyanidin-3-rutinoside, respectively, which were higher than those found in yellow tropical fruits. The quality of both purple and white açaí varied significantly among mesoregions, with fruits from floodplain soils demonstrating superior quality compared to those from sandy and solid soils in southeastern Pará. Mineral composition and microbiological characteristics were similar between white and purple açaí. These findings underscore the significant influence of mesoregion and soil type on açaí quality, emphasizing the superiority of fruits grown in floodplain soils.

## 1 Introduction

Açaí tree (*Euterpe oleracea* Mart.) is a native palm type from the Amazon that produces the purple açaí fruit, which is the best known and most studied variety. The white açaí is an ecotype that occurs naturally among populations of açaí species along the Amazon estuary; its mature fruits are opaque green and its drink is greenish or light yellow. There are many differences between the two types, and the white variety is still unknown in the scientific literature (1, 2). The white açaí, still little studied, is an alternative as a sustainable management of açaí plantations with new technological options (alternative appeared twice), financially favoring the rural communities and the growth of the industrial market (3).

The large amount of açaí extraction in northern Brazil, in particular in the State of Pará, is explained in part by geographic factors, that is, areas of extensive floodplains, that favor the cultivation of açaí trees, and also by its regional demand that historically has been an essential food in the diet of the local population (4).

The area of Pará State is equivalent to 16% of the Brazilian territory area. About half of this percentage is influenced by tides, which, therefore, form part of the Union’s patrimony, totaling 8.5 million hectares of floodplain and island areas (5), highlighting the Marajó Archipelago. The seasonality of açaí fruits is a consequence of the extractive production way, which depends more heavily on climatic conditions and natural cycles, such as the transport of nutrients by rivers (6) and rain cycles.

**Figure 1:**
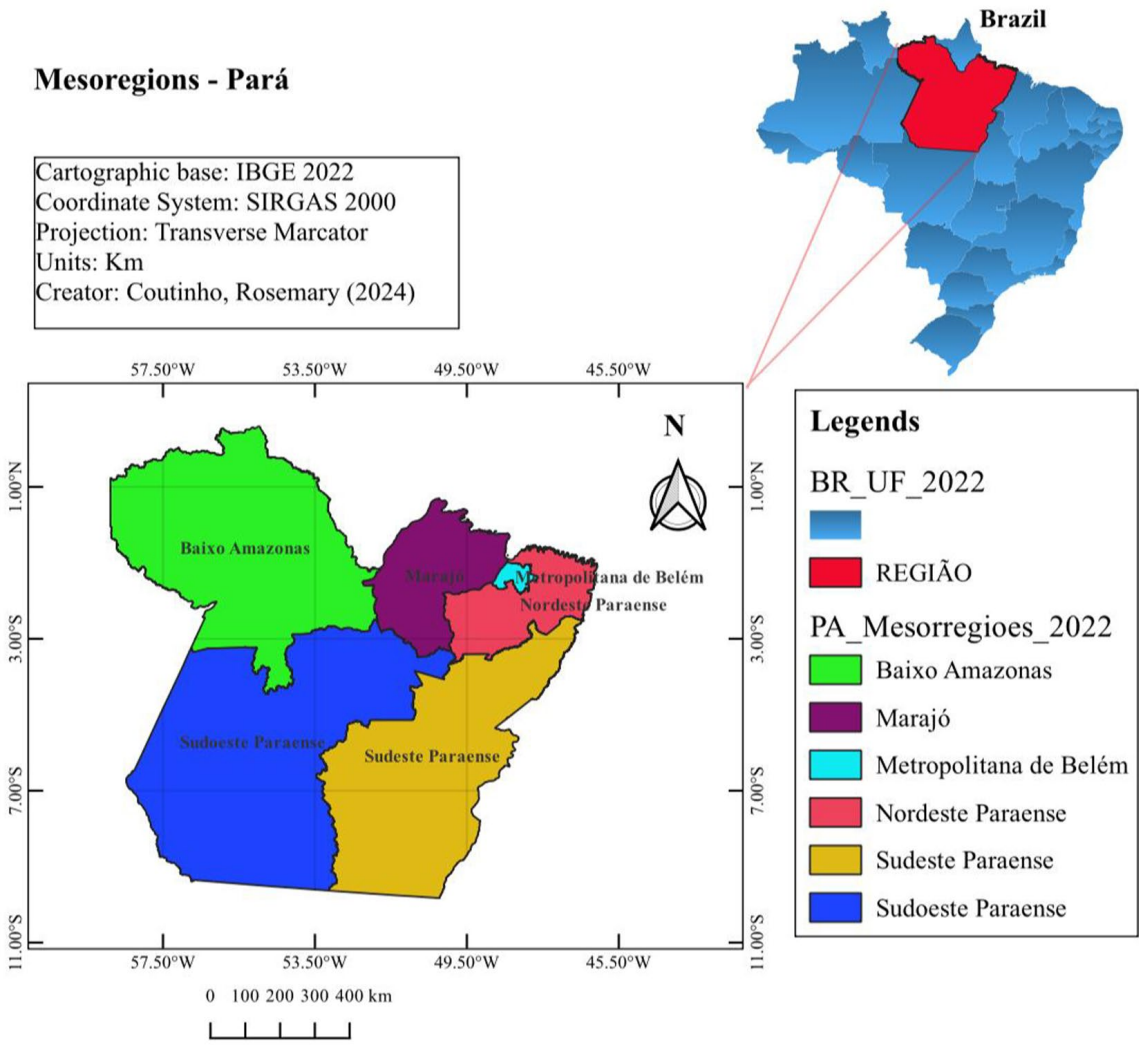
Map of the origin areas of purple and white açaí fruit in Pará State, that is, Marajó Island (Arari), the Northeast of Pará (Cametá), Metropolitan Belém (Benevides), the Southeast of Pará (Marabá), and Low Amazonas (Santarém).

The açaí from the islands, including the islets near Belém and Marajó Island, is considered an organic product, because it is totally derived from the extractive activity, fruit collected directly from the native açaí tree, with one of the best sensorial qualities, according to the local consumers, who prefer it over açaí from solid soil (7). The Northeast of Pará is the largest producing region of açaí (planted and native), followed by Marajó Island, Metropolitan Belém, Low Amazonas, and the Southwest and Southeast of Pará State (Figure 1). Most açaí production in the Metropolitan Region of Belém comes from cultivated or planted areas. The microregion of Cametá stands out in the mesoregion of Northeast Pará, which produced 43,048 tons of açaí in 2011, representing 39% of the state’s total production (8).

Currently, açaí is extensively studied by researchers worldwide and it is used in the food market as well as in the pharmaceutical and cosmetic industries due to its health benefits. These benefits are linked to its chemical composition, which is rich in phenolic compounds and anthocyanins. The antioxidant capacity of açaí is evaluated through various tests, including *in vitro* free radical sequestration. The fruits of the species *E. oleracea* and *E. precatoria* contain approximately 90 bioactive substances, with 31% being flavonoids, 23% phenolic compounds, 11% lignoids, and 9% anthocyanins, among others (9). Anthocyanins are glycosides of anthocyanidins belonging to the class of flavonoids and have the 4-hydroxyflavilium ion at its basic nucleus. They have been characterized as compounds responsible for the antioxidant activity of the purple açaí (10–12).

In recent years, açaí has benefited from its widespread use across various industries, including food and cosmetics. Its increasing popularity has led to its inclusion in the Botanical Dietary Supplements market, where it now ranks among the top 40 in the USA. Concurrently, cancer patients are increasingly adopting açaí as a complement to conventional chemotherapy (13–15). In this context, given the limited study of white açaí in the literature, it becomes essential a comparison of it to the purple açaí, whose functional properties are globally recognized.

The objective of this study was to identify and quantify monomeric anthocyanins using HPLC-MS from white açaí fruits, and compare the chemical, mineral, and microbiological characterization of two types of açaí fruits, white and purple, originated from five Brazilian Amazonic mesoregions.

## 2 Materials and methods

### 2.1 Collection

**Figure 2:**
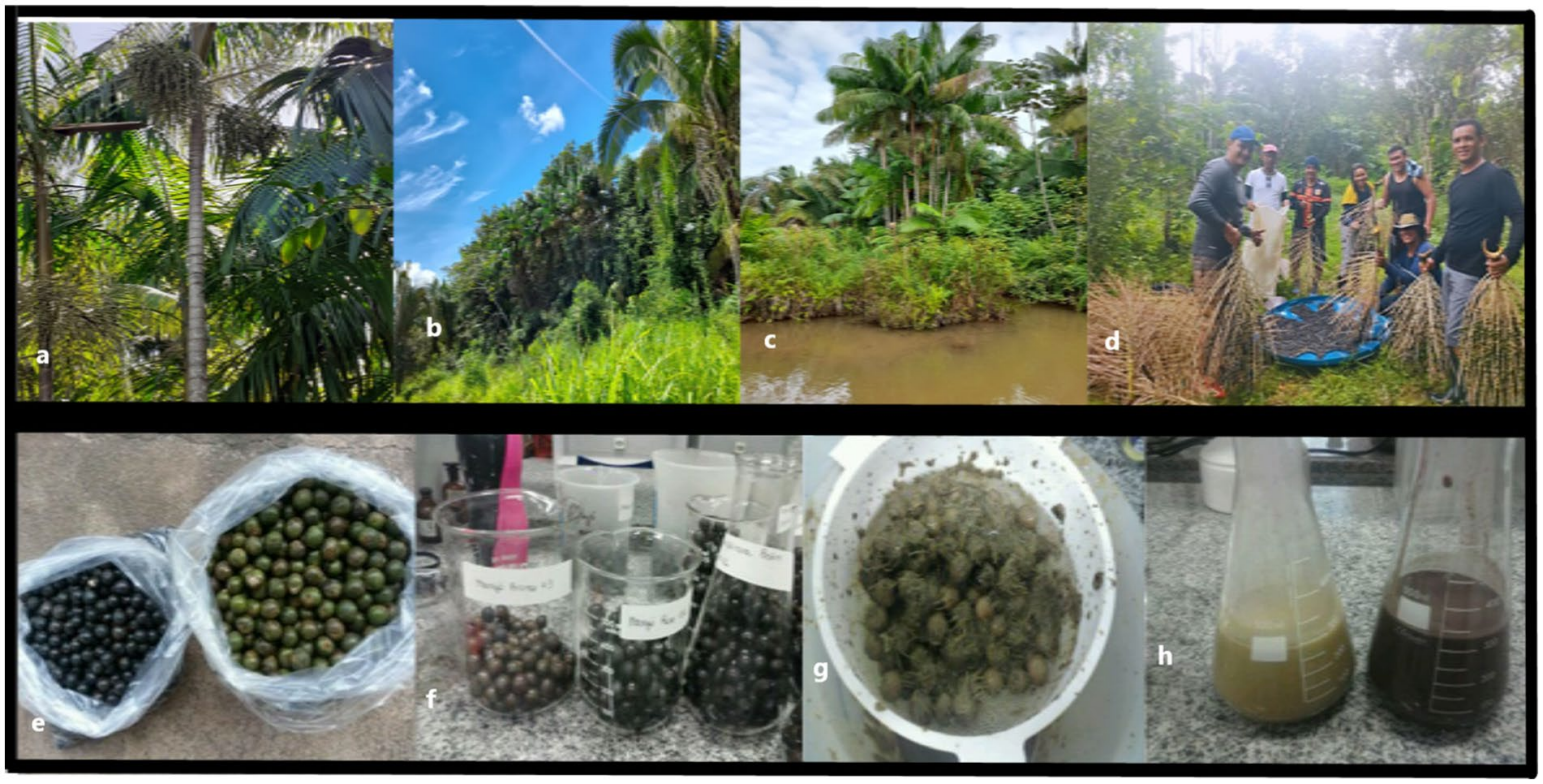
Native ecotype palm tree from Amazon, the açaí Tree (*Euterpe oleracea* Mart.) **(A)** Açaí palm (*Euterpe oleracea* Mart.). **(B)** Açaí production in agroforestry dry systems. **(C)** Floodplain forest. **(D)** Horseback journey for fruit harvesting in the Southeastern Pará Mesoregion. Mini-scale process of açaí beverage extraction: **(E)** Açaí seed with pulp **(F)** Sorting and immersion in warm water **(G)** Maceration and extraction through simple filtration; **(H)** White and purple açaí beverages.

The purple and white açaí fruits were collected from five Brazilian Amazonic mesoregions, such as Marajó Island, the Northeast of Pará, Metropolitan Belém, the Southeast of Pará, and Lower Amazonas (Figure 1). To represent the profile of the açaí fruit, five samples of purple and white açaí, each one weighing 3 kg, were collected from each mesoregion (Figure 2). in the vessels that transport the açaí from the country areas of the state to the city’s capital, at 4:00 a.m., at the Feira Ver-o-Peso peer, in Belém City, except for the samples from the Southeastern Pará (Marabá municipality) and Low Amazonas (Santarém municipality), which were collected in the respective municipalities.

The collected açaí fruits were packed in sterile plastic bags of polyethylene, and immediately packaged in a styrofoam box, the açai was kept in dry ice, and transported by airplane to Belo Horizonte City, Minas Gerais State, and by land to the Laboratory of Dyes and Bioactive Compounds of the Federal University of Viçosa, Viçosa City, Minas Gerais State. Until the time of analysis, the samples were stored in a freezer at −18°C temperature. All analyzes were performed in triplicates.

### 2.2 Physicochemical analysis

For the physicochemical analysis, 2 kg of each sample of purple and white açaí fruits were washed in the water, subjected to a 2% sodium hypochlorite solution for 20 min, rinsed with running water, and then soaked in water at approximately 45°C to soften the peel, followed by manual maceration at a ratio of 2:1 (fruit). The seeds were then separated by a vacuum filtration. A pasty violet liquid was obtained from the purple açaí and a pasty moss-green liquid from the white açaí. Portions of both liquids were used for the analysis of physicochemical characteristics, including Total Titratable Acidity (TTA), expressed as a percentage of citric acid, pH measured with a digital pH meter (DIGIMED DM-20), and Total Soluble Solids (TSS) determined by direct reading on a digital refractometer (Digital Hand-Held Refractometer AR200, Leica), expressed in °Brix. All analyses followed the analytical procedures proposed by the Adolfo Lutz Institute (ALI) (16). The analyses for TSS and crude fiber (*CF*) adhered to AOAC methodology (17). The contents of Reducing Sugars (RS) and Non-Reducing Sugars (NRS) were expressed in g/100 g of açaí fruit, according to the Somogyi-Nelson methodology (18).

### 2.3 Mineral content in the white açaí compared to the purple açaí

The phosphorus (P) content was determined by spectrophotometry and read at 724 nm; the contents of Calcium (Ca), Magnesium (Mg), Zinc (Zn), Copper (Cu), Manganese (Mn), and Iron (Fe) were analyzed by atomic absorption spectrometry (Varian model SpectrAA 22FS, Australia); and the Potassium (K) content was measured by photometry (Corning model 400, USA). All analyses were performed according to the Laboratory’s plant Analysis protocol, from the Department of Soils at the Federal University of Viçosa, MG, Brazil, following the methodology employed by Defelipo and Ribeiro (19). Results were expressed in mg/100 g of açaí fruit on a dry basis.

### 2.4 Chemical analysis

To determine total phenolics, total anthocyanins, and antioxidant capacity, ethanolic extracts were obtained from 200 g of purple and white açaí fruits. The weighed fruits were mixed in 200 mL of 70% ethanol, then acidified with concentrated hydrochloric acid (HCl) until reaching pH 2.0. The extracts were kept refrigerated for 48 h in the absence of light, then filtered under vacuum and centrifuged at 6,222 × *g* for 15 min, and concentrated in a rotary evaporator (IKA brand, model HB10) to approximately 5 mL.

The quantification of total phenolics was performed by the Folin-Ciocalteau spectrophotometric method, described by Singleton and Rossi (20), and the results were expressed in mg of gallic acid equivalent per gram of the extracts (mg/GAE/g). Total anthocyanins were quantified according to the method described by Lees and Francis (21), expressed in mg of anthocyanins per 100 mg of the fruit, using the average glycoside extinction coefficient (98.2 L/cm/g), corresponding to 3-glycoside (22). The determination of antioxidant capacity was performed using the Trolox equivalent antioxidant capacity (TEAC) assay, using the 2,2′-azino-bis 3-ethylbenzothiazoline-6-sulfonic acid (ABTS) radical, according to the methodology described by Re et al. (23). The results were expressed in μmol/L/Trolox/g per mL of sample.

### 2.5 Identification and quantification of anthocyanins in the white açaí fruit in HPLC-MS

The profiles of anthocyanins in the white açaí fruit extract were analyzed by HPLC-MS in the Enology and Natural Products Laboratory from the Castilla-La Mancha Regional Institute of Applied Scientific Research University, Spain. The used methodology was adapted from previously described methods by Castillo-Muñoz et al. (24). The identification was based mainly on spectroscopic data [UV–vis and MS/MS (Visible Spectroscopy and Mass Spectrometry)] obtained from authentic standards. Other method modified by de Brito et al. (25) was used for quantification of the same anthocyanins through a 5 μm C18 column (250 × 4.6 mm) in reverse phase and UV–Visible detectors (Shimadzu SPD-10 AV lamp) and diode arrays (DAD-Shimadzu, SPD-M20A).

### 2.6 Microbiological analysis

Portions of 100 g of both purple and white acai fruits were separated for microbiological analyses, preserving their natural sampling state. Rapid methods were employed using Petrifilm^®^ AC and EC plates following the manufacturer’s recommendations to enumerate mesophilic aerobes and *Escherichia coli*, respectively, according to Wehr and Frank (26). The Staph Express method (3 M Microbiology, St. Paul, MN, USA) was used for the analysis of coagulase-positive *Staphylococcus aureus* according to AOAC (27). The analysis for *Salmonella* sp. was carried out following the methodology described in ISO 6579. The results were expressed as the number of Colony-Forming Units per gram (CFU/g).

### 2.7 Statistical analysis

Data obtained from the chemical and physicochemical analyzes were interpreted by variance analysis (ANOVA), applying the F test at 5% probability level. To verify the difference among the means of the treatments (mesoregions, *n* = 5), the Tukey test was applied at 5% probability level, using the Statistical Analysis System (SAS) program, version 9.1, licensed by the Federal University of Viçosa.

In the revised methodology, a multifactorial statistical model was developed to investigate the differences between two varieties of açaí fruits, purple and white, and their relationship with different mesoregions. Factor 1 considered the two fruit varieties as independent variables, while Factor 2 represented the geographic mesoregions where the samples were collected. Each mesoregion was characterized by its environmental and agricultural management conditions. The statistical analysis included tests to assess data normality and homogeneity variance, along with comparisons of means using ANOVA and appropriate post-hoc methods. This approach facilitated exploring how regional conditions influence nutritional and quality characteristics of the studied açaí fruits.

## 3 Results and discussion

### 3.1 The comparison of physicochemical characteristics between the purple and white açaí from different mesoregions in Pará

The Table 1 presents a statistical analysis of different soil parameters in various regions of Pará, Brazil. These parameters include proteins (P^1^), pH (pH^2^), total titratable acidity (TTA^3^), total soluble solids (TSS^4^), crude fiber (CF^5^), reducing sugars (RS^6^), and non-reducing sugars (NRS^7^). The P1 of purple açaí ranged from 7.99 ± 0.07 to 13.91 ± 0.20, with the lowest values in “Low Amazon, Floodplain area” and the highest in “the Southeast of Pará wich has Solid and sandy soil” the white açaí, ranged from 9.42 ± 0.16 to 13.22 ± 0.45, with the lowest values in “the Northeast of Pará, Floodplain area” and the highest in “the Southeast of Pará, which has Solid and sandy soil.” The protein values were significantly different among regions and soil types, indicating an influence of the cultivation environment on protein concentration, suggesting that nutrient-rich soils tend to promote higher protein concentrations in açaí fruit.

**Table 1:** Comparison of physicochemical characteristics between purple and white açaí from different mesoregions in Pará.

Origin/Meso-Regions/Soil Type						
Analyzis	Type	Marajó Island Floodplain area	Northeast Pará Floodplain area	Metropolitan Belém cultivated	Southeast Pará solid and sandy soil	Low Amazon Floodplain area
P^1^	Purple	10.23 ± 0.26^a^	13.88 ± 0.33^c^	11.37 ± 0.19^b^	13.91 ± 0.20^c^	7.99 ± 0.07^d^
	White	12.83 ± 0.07^bc^	9.42 ± 0.16^a^	11.10 ± 0.75^ab^	13.22 ± 0.45^c^	12.55 ± 1.04^cb^
pH^2^	Purple	5.13 ± 0.03^a^	5.09 ± 0.04^a^	4.92 ± 0.0.03^b^	5.09 ± 0.01^a^	5.17 ± 0.09^a^
	White	5.39 ± 0.06^a^	5.49 ± 0.08^a^	5.32 ± 0.04^a^	5.30 ± 0.11^a^	5.51 ± 0.02^a^
TTA^3^	Purple	0.12 ± 0.01^c^	0.14 ± 0.00^bc^	0.15 ± 0.01^abc^	0.18 ± 0.02^ab^	0.18 ± 0.01^a^
	White	0.10 ± 0.00^a^	0.12 ± 0.03^a^	0.09 ± 0.01^a^	0.11 ± 0.03^a^	0.14 ± 0.01^a^
TSS^4^	Purple	3.35 ± 0.08^b^	4.25 ± 0.04^a^	3.50 ± 0.23^b^	2.49 ± 0.12^c^	3.33 ± 0.17^b^
	White	3.04 ± 0.29^b^	2.84 ± 0.47^b^	2.57 ± 0.19^b^	1.56 ± 0.11^c^	4.72 ± 0.13^a^
CF^5^	Purple	5.54 ± 0.24^d^	8.65 ± 0.11^b^	7.69 ± 0.24^c^	5.62 ± 0.17^d^	11.86 ± 0.41^a^
	White	8.03 ± 0.55^a^	4.41 ± 0.38^c^	6.20 ± 0.12^b^	7.78 ± 0.17^a^	4.14 ± 0.19^c^
RS^6^	Purple	0.79 ± 0.04^a^	0.30 ± 0.15^b^	0.73 ± 0.07^a^	0.99 ± 0.05^a^	0.81 ± 0.06^a^
	White	0.81 ± 0.04^b^	0.86 ± 0.02^b^	0.66 ± 0.02^c^	0.53 ± 0.02^d^	1.49 ± 0.04^a^
NRS^7^	Purple	0.25 ± 0.02^a^	0.08 ± 0.04^c^	0.17 ± 0.02^b^	0.24 ± 0.01^a^	0.25 ± 0.04^a^
	White	0.20 ± 0.02^b^	0.22 ± 0.02^b^	0.12 ± 0.01^c^	0.10 ± 0.01^c^	0.50 ± 0.12^a^

1 Proteins.

3 Total titratable acidity.

6 Reducing sugar.

7 Non-reducing sugar expressed as g 100 g^−1^.

4 Total soluble solids, expressed in °Brix.

5 Crude Fiber expressed in % and hydrogenionic potential.

2 Data expressed as mean of triplicate ± standard deviation. Means followed by the same letter in the rows do not differ at 5% probability by Tukey test. In the two-factor ANOVA test, each cell of the table contains the mean values of the contents for each combination of mesoregion and açaí variety, allowing the assessment of not only the main effects of the factors (variety and mesoregion) but also their interaction.

The pH in the purple açaí had similar values, ranging from 4.92 ± 0.03 to 5.17 ± 0.09. Very close to the white açaí, ranging from 5.30 ± 0.11 to 5.51 ± 0.02. The pH remained relatively stable across regions, which may indicate uniform soil acidity in different areas of Pará. This is consistent with other studies suggesting that pH variation in tropical soils tends to be limited. In the TTA^3^ analysis, the purple açaí ranged from 0.12 ± 0.01 to 0.18 ± 0.02, and the white açaí ranged from 0.09 ± 0.01 to 0.14 ± 0.01. Titratable acidity showed variations among different regions, reflecting possible differences in the organic composition of the soil, suggesting that more acidic soils can influence nutrient availability and the açaí fruit growth. In TSS^4^, purple açaí varied significantly, from 2.49 ± 0.12 to 4.25 ± 0.04, and for the white açaí, from 1.56 ± 0.11 to 4.72 ± 0.13. The variation in TSS4 indicates differences in the amount of sugars and other soluble compounds present in the soil, which can be affected by soil fertility and cultivation type. For CF^5^, purple açaí presented values from 5.54 ± 0.24 to 11.86 ± 0.41, and white açaí varied from 4.14 ± 0.19 to 8.03 ± 0.55. Crude fiber showed a wide variation, suggesting differences in the structural composition of plants cultivated in different soils. More compact soils or those with lower nutrient availability can influence fiber concentration. For RS^6^, in purple açaí, the values were relatively constant, from 0.30 ± 0.15 to 0.99 ± 0.05, and in white açaí, they varied from 0.53 ± 0.02 to 1.49 ± 0.04. Reducing sugars varied among regions, reflecting possible differences in plant maturation and sugar metabolism. For NRS^7^, in purple açaí, values ranged from 0.08 ± 0.04 to 0.25 ± 0.04, and in white açaí, from 0.10 ± 0.01 to 0.50 ± 0.12. The variation in non-reducing sugars indicates different cultivation conditions and possible agricultural practices influencing sugar composition in açaí fruit cultivation.

The results showed significant variations in the analyzed parameters among different regions and soil types in Pará. These differences can be attributed to factors such as soil composition, management practices, and climatic conditions. The comparison with other studies reveals consistency with existing literature, indicating that soils with different physicochemical characteristics influence the chemical composition of açaí fruits in diverse ways. These results emphasize the importance of not only considering the açaí variety but also the geographical and environmental context when assessing its nutritional composition.

To perform the statistical analysis of the physicochemical data, a two-factor analysis of variance (ANOVA) was conducted. The model structure was determined as Factor 1 [Fruit variety (purple vs. white)], Factor 2 [Mesorregion (Marajó Island, Northeast Pará, Metropolitan Belém, Southeast Pará, Low Amazon)], and [Dependent variables (P^1^, pH^2^, TTA^3^, TSS^4^, CF^5^, RS^6^, NRS^7^)]. This model allowed us to verify if there was an interaction between the factors and which factor had a significant effect on the analyzed variables. The results (Table 2) indicated whether there were significant effects of the factors and their interactions. The significances were checked based on the *p*-values.

**Table 2:** Results of the two-factor ANOVA analysis.

Variable	Factor	F	*p*-value	Significances	Results
P^1^	1	6.45	<0.05	S	The effect of variety varies according to the mesoregion.
	2	11.32	<0.001	Vs	
	3	3.87	<0.05	S	
pH^2^	1	8.21	<0.01	S	A consistent influence of fruit variety regardless of the mesoregion.
	2	7.43	<0.01	S	
	3	2.67	>0.05	Ns	
TTA^3^	1	5.62	<0.05	S	Variations in the influence of fruit variety depending on the mesoregion.
	2	9.87	<0.001	Vs	
	3	4.12	<0.05	S	
TSS^4^	1	12.34	<0.001	Vs	Variations in the influence of fruit variety depending on the mesoregion.
	2	14.56	<0.001	Vs	
	3	5.23	<0.01	S	
CF^5^	1	7.89	<0.01	S	The influence of the variety varies according to the mesoregion.
	2	10.02	<0.001	Vs	
	3	3.98	<0.05	S	
RS^6^	1	9.15	<0.01	S	Variations according to the mesoregion.
	2	8.67	<0.01	S	
	3	4.45	<0.05	S	
NRS^7^	1	11.87	<0.001	Vs	Variations in the influence of fruit variety depending on the mesoregion.
	2	12.76	<0.001	Vs	
	3	6.34	< 0.01	S	

1 Fruit variety (purple vs. white).

2 Mesoregion (Marajó Island, Northeast Pará, Metropolitan Belém, Southeast Pará, Low Amazon).

3 Interaction between fruit variety and mesoregion. Significance: (S) Significant e (Vs) Very significant; (Ns) Not significant.

The most significant results of the two-factor ANOVA statistical analysis (Table 2) showed that fruit variety (purple vs. white) and mesoregion (Marajó Island, Northeast Pará Lowland, Metropolitan Belém, Southeast Pará, Low Amazon) had significant effects on all analyzed variables (P, pH, TTA, TSS, CF, RS, and NRS). The interactions between the factors were also significant for most variables, indicating that the effects of fruit variety depended on the mesoregion. For example, protein (P) was influenced by both variety and mesoregion (*F* = 6.45, *p* < 0.05 and *F* = 11.32, *p* < 0.001, respectively), with a significant interaction (*F* = 3.87, *p* < 0.05), while pH was influenced by the same factors but without significant interaction. These complex interactions suggested that the açaí nutritional composition is highly dependent on geographic location and fruit variety. The concentration of the analyzed variables presented a complexity according to the combination of variety and location. These variations indicated that the açaí quality and nutritional value can be optimized by carefully selecting varieties and adapting agricultural practices to specific regional conditions, providing an opportunity to improve the production and açaí sales based on its desired properties. The *F* values and their associated *p*-values indicated that both açaí fruit variety and mesoregion had significant effects on the analyzed variables. The significant interaction between the factors in several variables highlighted the need to consider both factors when optimizing agricultural practices and improving the quality of the fruits under study.

**Figure 3:**
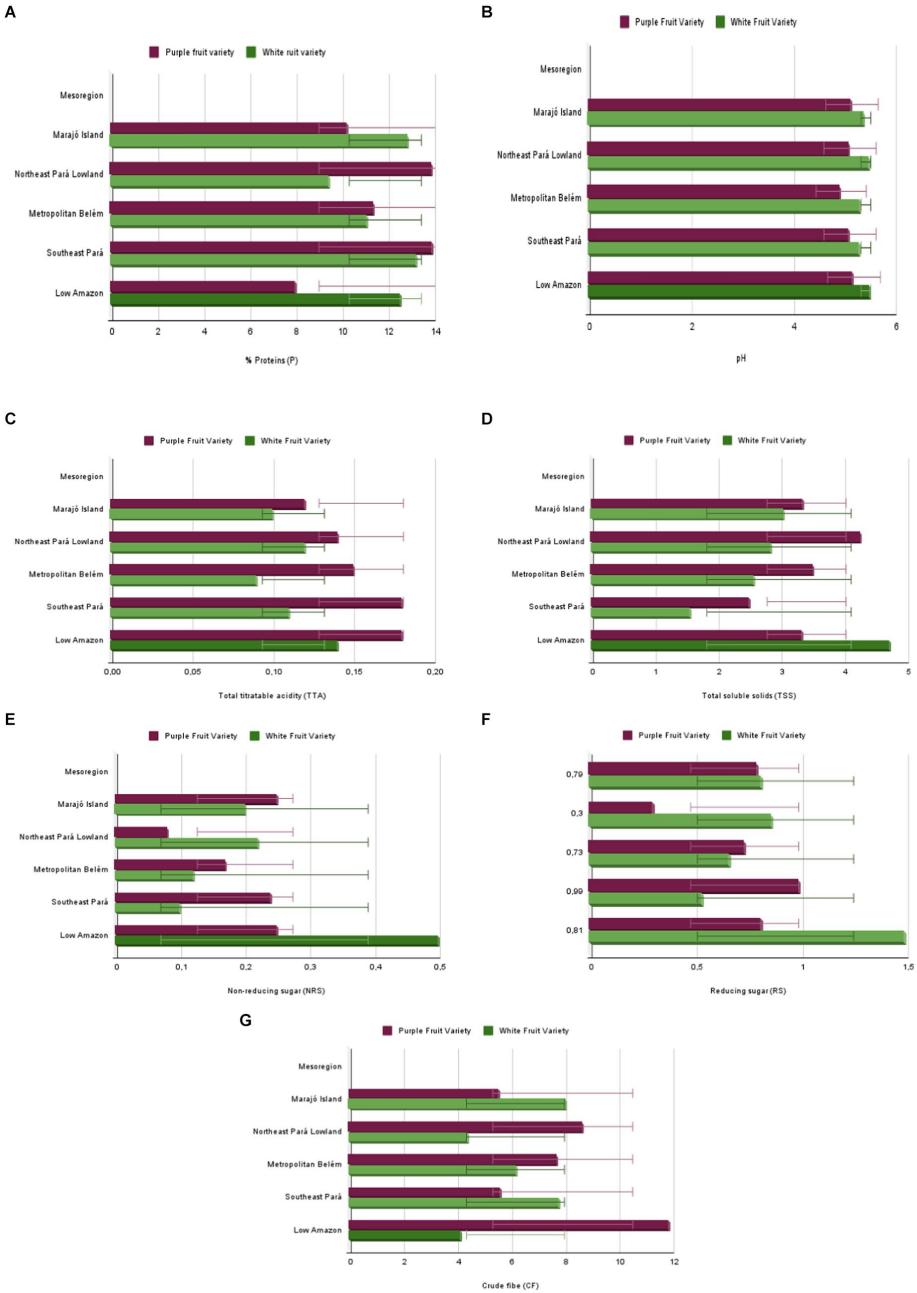
Graphical analysis of the physicochemical properties **(A)** P^1^, **(B)** pH^2^, **(C)** TTA^3^, **(D)** TSS^4^, **(E)** CF^5^, **(F)** RS^6^, **(G)** NRS^7^ of Purple and White Açaí Fruit from Pará’s Mesoregions.

The physicochemical results of the purple and white açaí fruits from various regions of Pará showed significant differences, which can be visualized in bar graphs (Figure 3). Proteins (a. P^1^) varied widely, indicating soils richer in nutrients. The b. pH^2^ remained stable, suggesting uniform soil acidity. Titratable acidity (c. TTA^3^) exhibited variations, reflecting differences in the organic composition of the soil. Total soluble solids (d. TSS^4^) also varied, indicating differences in soil fertility. Crude fiber (e. CF^5^) and reducing sugars (f. RS^6^) and non-reducing sugars (g. NRS^7^) showed regional variations, highlighting the influence of cultivation conditions and agricultural practices.

**Figure 4:**
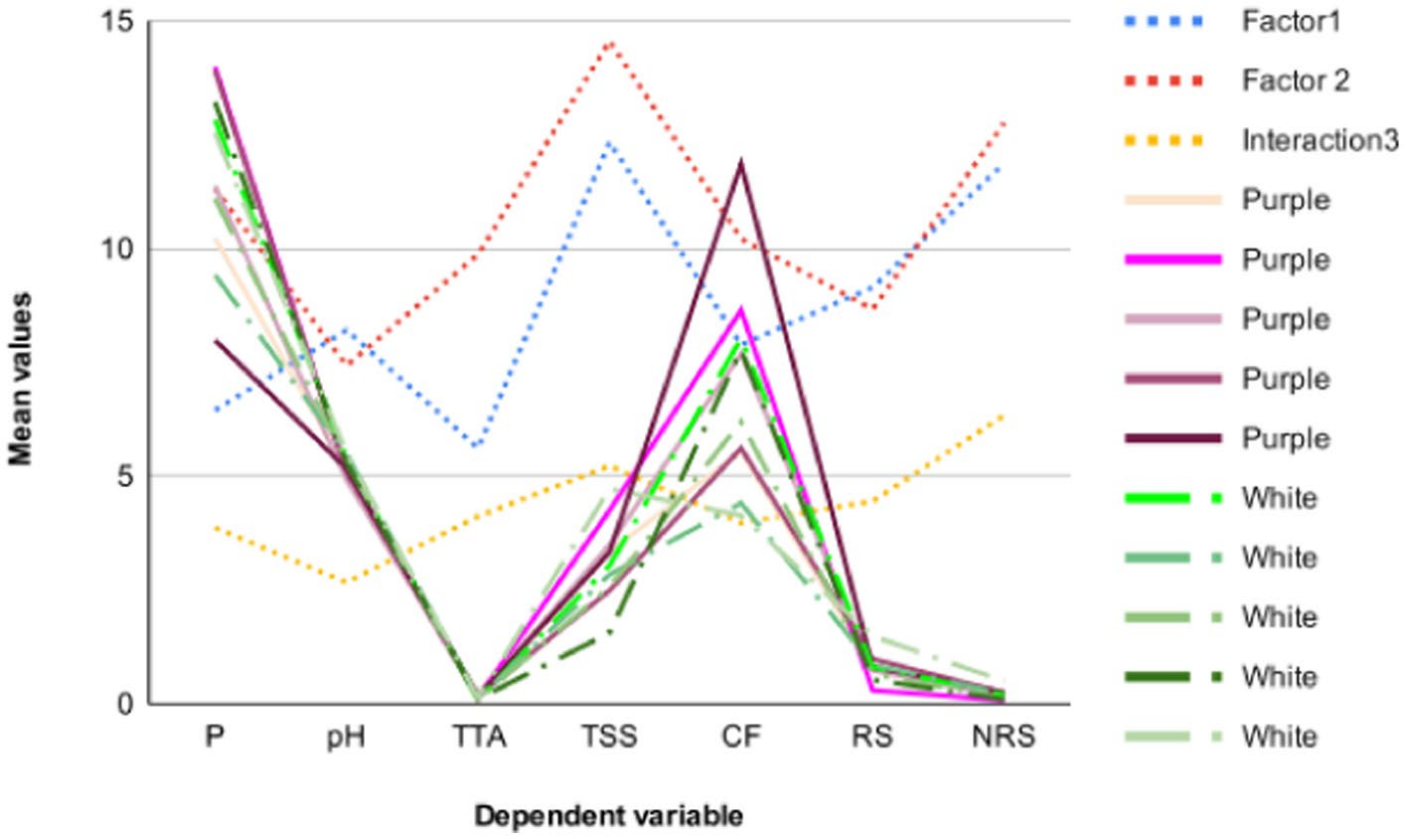
Graphical analysis from the interactions of the means of the dependent variables among different mesoregions for each variety of the açaí fruit. Being Factor 1 (purple and white fruit), Factor 2 (mesoregion), and Interaction 3 (Marajó Island Lowland, Northeast Pará Lowland, Metropolitan Belém Cultivated, Southeast Pará Solid Soil, Low Amazon Lowland).

The interaction plot (Figure 4) illustrates the mean values of physicochemical variables of white and purple varieties of açaí across different mesoregions. Each line represents the mean of a dependent variable (P—protein, pH, TTA—total titratable acidity, TSS—total soluble solids, CF—crude fiber, RS—reducing sugars, NRS—non-reducing sugars). Key observations include: Protein (P): The purple variety generally exhibited higher mean protein levels than the white variety in certain regions. pH: The white variety tended to have higher pH values overall compared to the purple variety. TTA: The purple variety showed higher acidity in some regions, while the white variety exhibited higher values in others. TSS: The purple variety reached higher peaks of TSS compared to the white variety, indicating greater soluble solids total in specific mesoregions. CF: The purple variety displayed a more pronounced peak in crude fiber compared to the white variety, especially in specific mesoregions. RS and NRS: Both varieties showed significant variations, with the white variety showing higher values of both sugars in some regions. The blue dashed line (Factor 1), red dashed line (Factor 2), and yellow dashed line (Interaction 3) indicated interactions and main effects of different factors. The variation between the green lines (white variety) and purple lines (purple variety) demonstrated that both the variety and mesoregion of açaí significantly influenced the fruit’s physicochemical properties. These specific patterns can guide agricultural and commercial practices, optimizing production according to the açaí desired characteristics.

This research highlights the nutritional and quality differences between the two types of açaí under different cultivation conditions. Açaí is recognized as an abundant source of bioactive compounds, such as antioxidants, vitamins, minerals, and essential fatty acids (28, 29). These elements are essential for health benefits associated with açaí consumption, including antioxidant, anti-inflammatory, and neuroprotective properties (29, 30). The physicochemical quality of açaí, including total solids content, pH, acidity, sugar content, fiber, and other components, can directly influence the concentration and activity of bioactive compounds present in the fruit. For example, the presence of high levels of antioxidants, such as anthocyanins and flavonoids, is related to a higher concentration of total solids and lower acidity of açaí. Similarly, the fruit proper preservation, through techniques such as freezing or freeze-drying, can help maintain the integrity of these bioactive compounds over time. Therefore, ensuring the açaí physicochemical quality and implementing proper preservation practices are essential to maintain the bioactive capacity of the fruit and, consequently, the health benefits associated with its consumption.

### 3.2 Mineral content in white açaí compared to purple açaí

**Figure 5:**
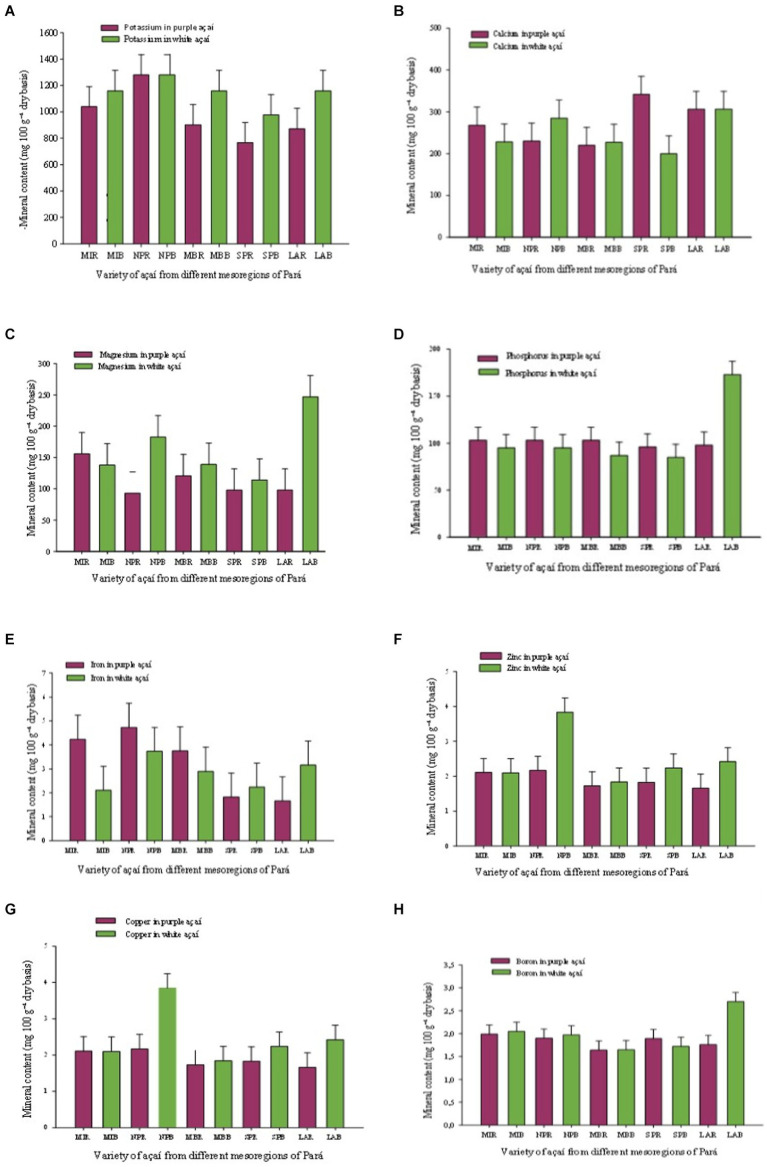
Averages and standard error of the mineral content comparison between purple açaí (R) and white açaí (B) fruits from different regions in Pará. Ilha de Marajó Island (MI), Northeast Pará (NP), Metropolitan Belém (MB), Southeast Pará (SP), and Lower Amazon (LA). **(A)** K, **(B)** Ca, **(C)** P, **(D)** Mg, **(E)** Fe, **(F)** Zn, **(G)** Cu and **(H)** B.

Comparing the white açaí to the purple açaí fruit, in relation to levels of mineral elements, the white açaí provided the best benefits of mineral content in this study. The K, Mg, Mn, P, Zn, and Cu levels were higher in the white fruit and the Ca, Fe, and B levels were similar in both varieties. When comparing fruits by mesoregions, the levels of mineral elements of the white fruits from Lower Amazonas (LA) and Northeast Pará (NP) were higher, and the purple fruits from Marajó Island (MI) presented higher levels (Table 3). This result also explains that floodplain soils have high fertility, likely due to the successive deposition of sediments, and a pH ranging from 4.5 to 5.5. However, solid soils are important options for cultivating this palm tree, but under conditions of low water deficiency, because the açaí plant is a typical species of floodable areas. The groundwater oscillations determine greater or less water and oxygen availability, provoking the processes of iron oxidation and reduction, responsible for the mottles’ emergence that characterizes these flooded soils (31). This fact means that the soil has less or greater amount of Fe, and consequently its presence in the açaí fruit. Thus, the iron content will vary, depending on the region in which the açaí was collected, whether in solid soil or lowland soil, and that is a likely explanation for the great variability of results reported in the literature. In this study, the iron contents indicated that fruits from mesoregions of the Northeast Pará (NP) (3.73 mg100/g), Low Amazonas (LA) (3.16 mg100/g), and Metropolitan Belém (MB) (2.90 mg100/g) were slightly higher when compared to fruits from Southeast Pará (SP) (2.24 mg100/g) and Marajó Island (MI) (2.11 mg100/g). White açaí had the highest potassium content (1,159 mg100/g, NP) in this study, and no data was found in the literature to compare it to the purple açaí (Table 3 and Figure 5).

**Table 3:** Means and standard deviation of the mineral content comparison of the purple (p) and white (W) açaí fruits, from different sources, in Pará.

Mineral	Type	Marajó Island Floodplain area	Northeast Pará Floodplain area	Metropolitan Belém cultivated	Southeast Pará solid and sandy soil	Low Amazon Floodplain area
Potassium (K)	Purple	1030.0 ± 0.37^b^	660.0 ± 0.26^b^	902.0 ± 0.45^b^	766.0 ± 0.55^b^	872.0 ± 0.82^b^
	White	1159.0 ± 0.27^a^	1280.0 ± 0.66^a^	1080.0 ± 0.84^a^	978.0 ± 0.73^a^	1159.0 ± 1.25^a^
Calcium (Ca)	Purple	268.0 ± 1.02^a^	230.0 ± 0.74^b^	220.0 ± 0.83^a^	342.0 ± 0.11^a^	155.0 ± 1.09^b^
	White	228.0 ± 0.96^b^	285.0 ± 0.88^a^	227.0 ± 0.14^a^	200.0 ± 0.11^b^	306.0 ± 2.02^a^
Phosphorus (P)	Purple	103.0 ± 5.01^a^	103.0 ± 5.04^a^	103.0 ± 1.02^a^	96.0 ± 0.49^a^	98.0 ± 1.11^b^
	White	95.0 ± 5.05^a^	95.0 ± 5.03^a^	87.0 ± 2.03^b^	85.0 ± 1.96^b^	173.0 ± 2.21^a^
Magnesium (Mg)	Purple	156.0 ± 3.08^a^	93.0 ± 4.14^b^	121.0 ± 3.23^b^	98.0 ± 2.19^b^	98.0 ± 2.07^b^
	White	138.0 ± 2.29^b^	183.0 ± 2.37^a^	139.0 ± 4.39^a^	114.0 ± 2.61^a^	247.0 ± 3.13^a^
Manganese (Mn)	Purple	64.8 ± 4.88^a^	12.2 ± 2.11^b^	78.5 ± 1.57^a^	53.7 ± 0.24^a^	12.6 ± 2.35^b^
	White	59.7 ± 5.51^a^	65.1 ± 3.39^a^	52.6 ± 1.22^b^	53.9 ± 0.17^a^	23.3 ± 2.15^a^
Iron (Fe)	Purple	4.2 ± 0.55^a^	4.7 ± 0.95^a^	3.8 ± 0.91^a^	1.8 ± 0.85^a^	1.7 ± 0.56^a^
	White	2.1 ± 0.67^b^	3.7 ± 1.12^a^	2.9 ± 1.01^a^	2.2 ± 0.52^a^	2.2 ± 0.54^a^
Zinc (Zn)	Purple	2.1 ± 0.13^a^	2.2 ± 0.14^b^	1.7 ± 0.35^a^	1.8 ± 0.73^a^	1.7 ± 0.74^a^
	White	2.1 ± 0.15^a^	3.8 ± 0.32^a^	1.8 ± 0.31^a^	2.2 ± 0.41^a^	2.4 ± 0.18^a^
Copper (Cu)	Purple	1.2 ± 0.22^a^	0.8 ± 0.71^b^	1.6 ± 0.89^a^	1.0 ± 0.43^a^	1.0 ± 0.33^a^
	White	1.1 ± 0.31^a^	2.2 ± 1.82^a^	0.9 ± 0.75^a^	1.1 ± 0.47^a^	1.9 ± 1.04^a^
Boron (B)	Purple	2.0 ± 1.22^a^	1.9 ± 2.78^a^	1.6 ± 1.69^a^	1.9 ± 0.31^a^	1.8 ± 1.33^a^
	White	2.1 ± 1.41^a^	2.0 ± 2.82^a^	1.7 ± 1.70^a^	1.7 ± 0.55^a^	2.7 ± 1.04^a^

Marajó Island (MI), Northeast Pará (NP), Metropolitan Belém (MB), Southeast Pará (SP), and Lower Amazon (LA). Data expressed as mean of triplicate ± standard deviation. Means followed by the same letter in the rows do not differ at 5% probability by Tukey test. In the two-factor ANOVA test, each cell of the table contains the mean values of the mineral contents for each combination of mesoregion and açaí variety, allowing for the assessment of not only the main effects of the factors (variety and mesoregion) but also their interaction.

When compared to other fruits popularly consumed in the Amazon region, like banana *prata* (*Musa* spp. *Musaceae* family) with 330 mg100/g; banana *nanica* (*Musa paradisiaca* L./*Musaceae* family) with 320 mg100/g; and passion fruit, with 338 mg100/g (32, 33), which can be considered good potassium sources, açaí mineral content level is three times higher, which suggests that the fruit is a good potassium (K) source. The calcium (Ca) content for the white açaí ranged from 200 mg100/g [Southeast Pará (SP)] to 306 mg100/g [Low Amazon (LA)]. The phosphorus (P), magnesium (Mg), and manganese (Mn) content was also significant in white açaí. The highest phosphorus (P) content was 173 mg100/g (LA) and the lowest was 85 mg100/g (SP). The magnesium content was higher in Low Amazon (247 mg100/g), in relation to Southeast Pará (114 mg100/g). The highest manganese content for white açaí and purple açaí was, respectively, 65.1 mg100/g (NP) and 78.5 mg100/g (MB). The superior quality of the manganese mineral in the açaí fruit, compared to other foods, distinguishes it as a natural food supplement. The minor contents of zinc (Zn), copper, and boron (B) were also quantified. Coutinho et al. (34), upon evaluating açaí pulp, reported that the consumption of this pulp, rich in these minerals, can help ensure the growth and proper functioning of the human body in general.

The graphs present (Figures 5A–H) a detailed analysis of the minerals found in purple and white açaí fruits across various regions of Pará. Significant variations were observed in the concentration of essential minerals such as calcium, iron, potassium, and magnesium among the analyzed regions. These differences can be attributed to soil composition variations, cultivation practices, and specific environmental factors of each area. The mineral diversity highlighted the complexity of interactions between soil and plant, providing valuable insights to maximize nutritional benefits and health advantages associated with açaí consumption.

### 3.3 Chemical characterization of white and purple açaí fruits (control) from different mesoregions in Pará

All chemical analysis results of purple and white açaí fruits (Table 4) differed significantly. The total phenol content (TP) of the ethanolic extracts obtained from the purple açaí fruit varied from 442.56 (Southeastern Pará) to 806.17 mg/GAE/100/g (Low Amazonas); and for the white açaí fruit, the variation was from 224.20 (Southeastern Pará) to 401.92 mg/GAE/100/g (Marajó Island), values less than those observed in the purple açaí fruit (Table 3). These white açaí values are higher than those found in grape fruits (35) and jambolan [*Syzygium cumini* (L.) Skells] (36). Cipriano (37) found total phenolic contents for the purple açaí extract with the value of 574 mg/GAE/100/g; Hogan et al. (38) found 312 mg/GAE/100/g and Kuskoski et al. (36) found 136.8 mg/GAE/100/g; the last two values were lower than those found in the purple açaí study; and for any comparison, no study was found until now for the white açaí. Phenolic compounds present in fruits and vegetables are some of the main responsible compounds for their antioxidant capacity (AC). Their final content can be influenced by factors such as maturation, species, cultivation practices, geographical origin, growth stage, harvesting conditions, and storage process (39). In this study, the variation of total phenolic content of fruits among the mesoregions was significant, influencing the total anthocyanin (TA) content and consequently the antioxidant capacity (AC).

**Table 4:** Comparison of chemical characteristics between purple and white açaí from different mesoregions in Pará.

		Origin/Mesoregions/Soil Type				
Analyzis	Type	Marajó Island Floodplain area	Northeast Pará Floodplain area	Metropolitan Belém cultivated	Southeast Pará Solid and sandy soil	Low Amazon Floodplain area
TP	Purple	647.61 ± 64.38^b,c^	796.41 ± 26.85^a,b^	742.56 ± 59.90^b^	442.56 ± 41.80^d^	806.17 ± 17.48^a^
	White	401.92 ± 52.70^a^	379.75 ± 65.26^a^	354.17 ± 26.50ª^,b^	224.20 ± 8.85^b,c^	241.06 ± 17.50^b,c^
TA	Purple	70.93 ± 4.24^b,c^	75.14 ± 6.12^b,c^	65.58 ± 6.52^c^	40.63 ± 2.71^d^	81.73 ± 1.77^a^
	White	37.70 ± 5.34^a^	35.47 ± 6.62ª^,b^	32.87 ± 2.68ª^,b^	16.46 ± 6.61^d^	22.70 ± 0.90^c^
AC	Purple	18.78 ± 0.55^a^	15.08 ± 1.23^b,c^	13.17 ± 1.31^c^	4.21 ± 0.85^d^	19.25 ± 0.35^a^
	White	6.17 ± 1.07^a^	5.22 ± 0.98^a^	5.88 ± 0.55^a^	1.03 ± 0.73^c^	3.56 ± 0.18^b^

Total phenolics (TP) (mg/GAE/100/g—wet basis), Total Anthocyanins (TA) (mg.100/g fruit—wb), Antioxidant Capacity (AC) (μmol/L Trolox/g) per mL of sample. Data expressed as mean of triplicate ± standard deviation. Means followed by the same letter in the rows do not differ at 5% probability by Tukey test.

The total anthocyanin concentration (TA) in the purple açaí ethanolic extract ranged from 40.63 (Southeastern Pará) to 81.71 mg/100/g (Low Amazon); and for the white açaí fruit, it ranged from 16.46 to 37.70 g/100/g. Yuyama et al. (40) found values with upper and lower limits ranging from 104.3 and 15.4 g/100/g, on a dry basis, in the white açaí from Parintins municipality (Amazonas State) and it was not possible to compare them with our data, that were performed on wet basis (wb) (Table 1). Cavalcanti et al. (41) reported TA contents of 3.16 for strawberry, 13.52 for grapes, and 18.45 for purple açaí, all expressed in g/100/g, and these values were lower than those found in this study of the white açaí fruit extracts. Cipriano (37) found values of 74.8 g/100/g for the purple açaí fruit on a wet basis. Rogez (42) quantified anthocyanins from sixty samples of purple açaí (*E. oleracea*) and found a fruit total anthocyanin (TA) mean content of 44 mg/100/g, values similar to those found in this study. Bobbio et al. (43) confirmed the potential of açaí as TA source, when they found 263 mg/100/g content in the açaí fruit bark. The great variation in TA content found by these authors and also in our study can be justified due to the higher or lower presence of pigments among açaí populations.

The anthocyanin content values found for ethanolic extracts of the white açaí fruit were lower than those of the purple fruit, in all evaluated mesoregions. The AC found in the extracts of purple açaí ranged from 4.21 (Southeastern Pará) to 19.25 μmol/L Trolox/g (Low Amazon). The antioxidant capacity (AC) found in the white açaí fruit extract ranged from 1.03 (Southeast Pará) to 6.17 μmol/L Trolox/g (Marajó Island), all obtained on a wet basis, by the ABTS radical (Table 4). Kuskoski et al. (36) found AC value of 6.9 μmol Trolox 100/g in the purple açaí; and Cipriano (37) found content of 11.71 μmol Trolox 100/g of AC in the purple açaí pulp, in wet basis. No results were found in the literature for the white açaí. Sun et al. (44) stated that the greatest contribution to the total AC of fruits is due to the composition of phytochemical compounds and their influence on the antioxidant activity, mainly the anthocyanin pigments. Kuskoski et al. (36) studied the antioxidant capacity of pulps in wild tropical fruits as fruits with yellow pulps, such as pineapple (0.6), mango (13.7), graviola (*Annona muricata*) (4.5), cupuaçu (*Theobroma grandiflorum*, *Sterculiaceae* family) (1.1), and passion fruit (1.02), all estimated in μmol Trolox/g. The values found in this study on the extracts of white açaí were in this range of values, showing a good antioxidant capacity, even being inferior to those found in the purple fruit extracts.

The results indicated that the purple variety of açaí exhibited significantly higher levels of bioactive compounds compared to the white variety, specifically in terms of total phenolics (TP), total anthocyanins (TA), and antioxidant capacity (AC). These findings suggest that the purple variety offers greater health benefits due to its higher antioxidant content. These studies are valuable for the food industry and agricultural producers in selecting açaí varieties with higher nutritional value, influencing cultivation practices and the development of functional products. The nutritional and quality differences between the types of açaí can be attributed to environmental and crop management conditions, which need to be considered for a more precise analysis. The greater functionality of the purple açaí compared to white, in terms of TP, TA, and AC, can be explained by its genetic composition that promotes the synthesis of anthocyanins and phenolics, resulting in higher antioxidant capacity. This variation may be related to the specific environmental conditions of each mesoregion. In Marajó Island, the humid equatorial climate and traditional agroforestry systems may favor the synthesis of bioactive compounds. In the Northeast of Pará, intensive management along with irrigation in an equatorial climate may optimize the production of anthocyanins and phenolics. In Belém’s Metropolitan Region, nutrient-rich alluvial soils and the hot, humid climate promote high levels of antioxidant compounds. In the Southeast of Pará, the tropical climate with a dry season requires careful irrigation management, which may influence the variation of TP and TA. Finally, in the Lower Amazon, the equatorial climate with high rainfall and clayey soils favors floodplain cultivation, promoting high levels of AC. Thus, this study suggests that the soil, climate, and vegetation conditions from different Brazilian Amazonic mesoregions can have a fundamental impact on the nutritional and functional quality of açaí varieties.

The Student’s *t*-test revealed a significant difference (*t* = 4.18, *p* < 0.05) between the TP means of the purple and white varieties. The purple variety exhibited a significantly higher TP mean compared to the white variety. The results (Table 5) indicated that the purple variety consistently had higher levels of TP, TA, and AC compared to the white variety. The mesoregion analysis could reveal which geographical areas produced fruits with superior chemical characteristics, which may be useful for guiding cultivation practices and raw material selection. Investigating the interactions between fruit varieties and mesoregions could provide insights into how specific environmental factors affect the chemical characteristics of the fruits, potentially influencing production and marketing strategies. Therefore, it can be stated that for both Total Antho1cyanins (TA) and Antioxidant Capacity (AC), there were significant differences between the means of the purple and white varieties.

**Table 5:** Results of the two-way ANOVA with factors 1 (Fruit Variety) and 2 (Mesoregion).

Variable	Factor	*F*-value	*p*-value	Significances	Result
TP	1	45.37	<0.0001	S	This suggests that both fruit variety and mesoregion have a significant effect on the measured variables (TP, TA, and AC)
	2	18.24	<0.0001	S	
	3	8.53	<0.0001	S	
TA	1	64.25	<0.0001	S	
	2	21.56	<0.0001	S	
	3	9.87	<0.0001	S	
AC	1	82.45	<0.0001	S	
	2	25.13	<0.0001	S	
	3	12.34	<0.0001	S	

Factors: 1. Fruit variety (purple vs. white). 2. Mesoregion (Marajó Island, Northeast Pará Lowland, Metropolitan Belém, Southeast Pará, Low Amazon). 3. Interaction between fruit variety and mesoregion. Significanse: (S) Significant e (Vs) Very significant; (Ns) Not significant.

With the purpose to distinguish them, Wycoff et al. (45) made the chemical and nutritional analysis of the purple and white açaí fruits (*Euterpe oleracea* Mart.), comparing their nuclear magnetic resonance (NMR) spectra, and observed differences between the two types as well as the differences in the fruit pulp composition, proven fact in this study, when the two types were characterized. When comparing the different mesoregions, the two types presented significant differences, probably due to the soil type, either of lowland (native) or cultivated (planted), that presented superior chemical composition, when compared to the fruits from solid sandy soil type.

### 3.4 Identification and quantification of anthocyanins from extracts of the white açaí fruit by chromatographic techniques

**Figure 6:**
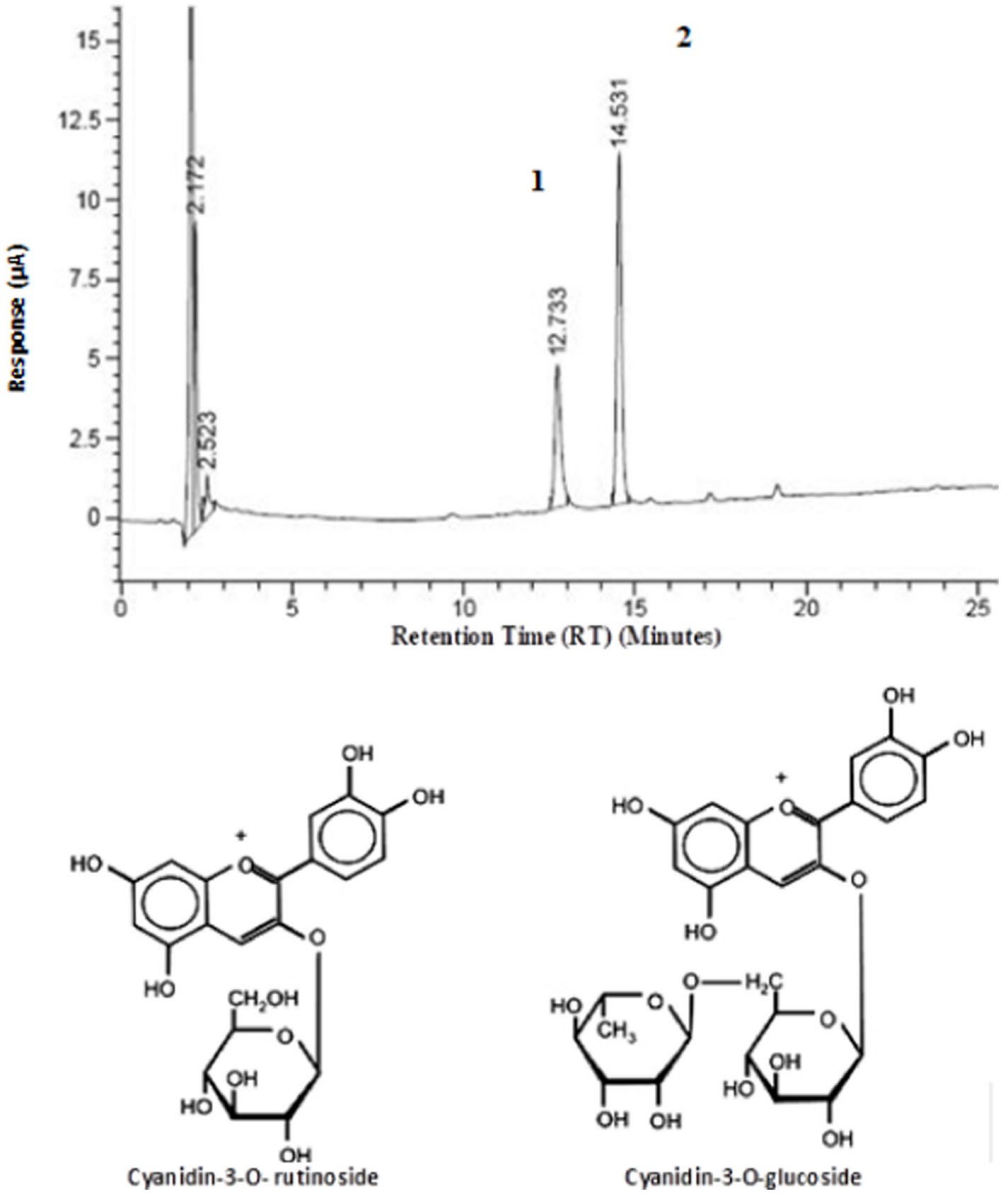
HPLC/MS chromatogram of the anthocyanin profile for the white açaí fruit extract. Cyanidin-3-O-rutinoside (peak 1) and Cyanidin-3-O-glucoside (peak 2).

High-performance liquid chromatography coupled with mass spectrometry (HPLC-MS) was employed using the modified methodology of de Brito et al. (25) and the adapted method from Castillo-Muñoz et al. (24) to identify and quantify anthocyanins in extracts of the white açaí fruit from different mesoregions in Pará. Table 6 presents the HPLC-MS data that identified and quantified the monomeric anthocyanins cyanidin-3-rutinoside and cyanidin-3-glucoside (Figure 6), confirming their presence in the white açaí fruit (*Euterpe oleracea* Mart.) (Figure 2H), a mutant variety of the purple açaí (Figures 2A–F). This study revealed that the two main anthocyanins found in the purple açaí extract are also present in the white açaí extract, albeit in lesser amounts. Further research is needed to identify other active components responsible for AC in the white açaí. The results highlighted significant variations in anthocyanin concentrations in the white açaí fruits collected from different mesoregions of the Brazilian Amazon. Table 6 displays the anthocyanin concentrations in the white açaí fruit extracts from various mesoregions of the Brazilian Amazon, analyzed using HPLC-MS methods A and B.

**Table 6:** Identification and quantification of anthocyanins isolated from the extract of the white açaí fruit.

Origin/Meso-regions	Peak*	RT^A^ (Min.)	RT^B^ (Min.)	[M]^+^(m/z)	MS–MS (m/z)	Concentration (μg 100 g^−1^) of fruit. Methods A and B	
Marajó Island/Floodplain area	1	12.7	12.6	449	287	0.59 ± 0.09	2.63 ± 0.03
	2	14.5	14.1	595	449–287	1.05 ± 0.10	3.13 ± 0.05
Northeast Pará/Floodplain area	1	13.0	13.4	451	289	0.51 ± 0.05	2.48 ± 0.08
	2	14.8	15.1	597	451–289	0.96 ± 0.07	2.97 ± 0.06
Metropolitan Belém/Cultivated	1	12.5	13.3	448	286	0.45 ± 0.08	1.51 ± 0.07
	2	14.3	15.8	594	448–286	0,91 ± 0.04	2.22 ± 0.05
Southeast/Solid and sandy soil	1	12.9	18.3	450	288	0.29 ± 0.12	1.01 ± 0.09
	2	14.6	20.1	596	450–288	0,74 ± 0.10	1.60 ± 0.02
Low Amazon/Floodplain area	1	12.8	19.5	452	290	0.35 ± 0.06	1.11 ± 0.04
	2	14.7	20.2	598	452–290	0,82 ± 0.03	2.10 ± 0.08

Peak*: Cianidina-3-glicosídeo^1^ and Cyanidin-3-rutinoside^2^, λ (520 nm). RT (retention times). molecular masses ([M]^+^), and MS–MS fragments.

The Table 4 results reveal significant variations in retention times (RT^A^ and RT^B^), molecular masses ([M]^+^), and fragments (MS–MS) of anthocyanins across different mesoregions of Pará. Each analyzed region exhibited unique profiles of chemical composition, as reflected by chromatographic peaks with good resolution and magnitude. Notably, peaks with RT^A^ between 12.5 and 14.8 min indicate the presence of two main anthocyanins: cyanidin-3-glycoside (peak 1) and cyanidin-3-rutinoside (peak 2), identified by molecular masses [M]^+^ of approximately 449–452 and 594–598, respectively. MS–MS fragments corroborated these identifications, with m/z 287 ions confirming the presence of cyanidin aglycone. Concentrations of anthocyanins ranged between 0.29 and 1.05 μg100/g for cyanidin-3-glycoside and between 0.74 and 3.13 μg100/g for cyanidin-3-rutinoside, depending on the analytical method (A or B), suggesting differences in extraction efficiency or method sensitivity. These findings highlight the complexity of the chemical composition of the white açaí and emphasize the importance of precise analytical methods for studying and assessing the nutritional value of this regional fruit. Results found by Wycoff et al. (45) differed from those of this study, when they stated in their analyzes that cyanidin-3-glucoside and cyanidin-3-rutinoside were not detected in the methanolic extract from 3.38 to 4.70% of the white açaí fruit, which, according to these authors, seems to differentiate even more the white açaí fruit, in relation to the purple fruit.

The Table 6 provides detailed data on retention times (RT^A^ and RT^B^), molecular masses ([M]^+^), and MS–MS fragments of two anthocyanin substances across various mesoregions of Pará. RT^A^ retention times varied slightly among regions, reflecting potential differences in sample matrices or specific analytical conditions at each location. Molecular masses [M]^+^ and MS–MS fragments also exhibit regional variations, indicating possible differences in the molecular composition of anthocyanins depending on the sample origin. Anthocyanin concentrations were generally higher when analyzed using method B, suggesting greater extraction efficiency or analytical sensitivity. These variations highlight the chemical complexity and diversity of anthocyanins in different Pará regions, influenced by local environmental factors and agricultural practices. Regarding white açaí being an ecotype of the purple açaí, the results suggest that regional chemical variations may be related to genetic or adaptive differences between these ecotypes. The white açaí, as a variant of the purple açaí, may exhibit lower and different profiles of anthocyanin contents, as reflected in observed [M]^+^ and MS–MS variations. This indicates that, while sharing a common ancestor, local adaptations and specific regional conditions can lead to notable differences in anthocyanin composition between the white and purple açaí, highlighting the genetic plasticity and adaptive capacity of açaí plants to diverse environments.

Hogan et al. (38) studied a purple açaí extract rich in anthocyanin by chromatographic analysis and identified three major anthocyanins: (a) peonidin-3- (6″-malonylglucoside), the most abundant, with RT of 13.20 min followed by cyanidin-3-rutinoside (11.34 min RT); (b) delphinidine 3- (6″-acetoyl) glucoside, with RT of 17.08 min; and (cyanidin-3-glycoside) (10.8 min RT). In another study by Lichtenthäler et al. (46), cyanidin-3-glucoside and cyanidin-3-rutinoside were determined as the main anthocyanins in açaí, whereas Bobbio et al. (10) observed that cyanidin-3-arabinoside and cyanidin-3-arabinosyl-arabinoside were the most abundant. In this context, it is important to note that other anthocyanins contribute to AC in the açaí fruit. In addition, there are still disparities between specific anthocyanins present in açaí.

Anthocyanins are pigments that impart purple or blue color to many fruits and flowers, and their presence can vary according to species and ecotype. In the case of the white açaí, despite its greenish hue, our studies have demonstrated a significantly lower amount of anthocyanins compared to the purple açaí. The difference in anthocyanin concentration between the white and purple açaí is likely attributed primarily to genetic and metabolic factors that regulate the production of these pigments. Literature reports indicate that there are not known natural populations of exclusively white açaí, but rather clusters (bunches) within purple populations. According to Tanaka et al. (47), such mutations can affect pigment production, such as anthocyanins, resulting in fruits with different colors, such as the opaque green of the white açaí. The major causes of these genetic mutations include errors in DNA replication, exposure to radiation, interaction with chemical agents, genetic recombination, transposable elements, natural selection, and genetic drift.

The white açaí is an alternative and less common variety of the purple açaí, which presents some differences in terms of color and flavor. Although less popular, it is gradually attracting interest due to its uniqueness and nutritional value in comparison with yellow fruits, as it may have potential health benefits.

### 3.5 Microbiological analysis

In all samples of the purple açaí fruit, the presence of total mesophiles was observed, with values ranging from 8.3 × 10^5^ CFU/g (Southeast Pará) to >3.0 × 10^6^ CFU/g (Marajó Island and Lower Amazon). Coliforms were present in all samples with values ranging from <10 CFU/g (Metropolitan Belém) to 1.0 × 10^3^ CFU/g (Marajó Island). In all samples, counts for *E. coli* were < 10 CFU/g. For filamentous fungi and yeasts, samples varied from 2.1 × 10^3^ CFU/g (Northeast Pará) to >3.0 × 10^5^ CFU/g (Marajó Island). The results for coagulase-positive *S. aureus* ranged from <10 CFU/g (Metropolitan Belém) to >1.5 × 10^5^ CFU/g (Lower Amazon). The presence of *Salmonella* sp. was detected in fruits from the Marajó Island, Northeast Pará, and Lower Amazon mesoregions (Table 7).

**Table 7:** Comparison of microbiological characteristics between the purple and white açaí from different mesoregions in Pará.

Origin	Type	Colony-Forming Unit per gram (CFU/g)					
		*Aerobic mesophilic*	*Escherichia coli*	Fungi and yeasts	*Staphylococcus. aureus*	*Salmonella* sp. (g)	Coliforms
Marajó Island/floodplain area	Purple	>3.0 × 106	<10	>3.0 × 10^5^	8.0 × 10^2^	Presence/25	1.0 × 10^3^
	White	>3.0 × 10^6^	<10	>3.0 × 10^5^	>3.0 × 10^5^	Presence/25	1.5 × 10^5^
Northeast Pará Floodplain area	Purple	1.3 × 10^6^	<10	2.1 × 10^3^	4.0 × 10^2^	Presence/25	7.0 × 10^2^
	White	>3.0 × 10^6^	<10	>3.0 × 10^5^	2.6 × 10^3^	Presence/25	<10
Metropolitan Belém cultivated	Purple	3.1 × 10^6^	<10	3.2 × 10^3^	<10	Absence/25	<10
	White	4.7 × 10^6^	<10	2.9 × 10^5^	>1.5 × 10^5^	Absence/25	>1.5 × 10^5^
Southeast Pará/Solid and sandy soil	Purple	8.3 × 10^5^	<10	1.1 × 10^5^	9.0 × 10^2^	Absence/25	5.0 × 10^2^
	White	>3.0 × 10^6^	<10	>3.0 × 10^5^	>1.5 × 10^5^	Absence/25	8.0 × 10^2^
Low Amazon/floodplain area	Purple	>3.0 × 10^6^	<10	1.2 × 10^4^	>1.5 × 10^5^	Absence/25	1.0 × 10^1^
	White	>3.0 × 10^6^	<10	1.2 × 10^5^	9.0 × 10^2^	Presence/25	<10

These microbiological results (Table 7) may indicate differences in cultivation methods, local environmental conditions, and açaí management practices, emphasizing the importance of food safety and the need for rigorous sanitary controls in the production and processing of açaí. Mudaliar et al. (48) highlighted that post-harvest loss in perishable products is a global concern, primarily attributed to infrastructure constraints during processing, handling, and storage. This loss is mainly influenced by microorganisms, which are the primary causes of post-harvest diseases in fruits and vegetables.

## 4 Conclusion

Despite having lower content in comparison with the purple açaí, white açaí fruits from the Northeast of Pará, Lower Amazon, and Marajó Island demonstrated superior quality in chemical and mineral characterization when compared to other tropical fruits. These fruits are classified as originated from floodplain areas. In contrast, fruits from the Southeast of Pará showed inferior results, as they come from solid soil areas. Our studies identified and quantified cyanidin-3-glucoside and cyanidin-3-rutinoside in the white açaí fruit extract, still relatively unknown in Brazil and globally, being one of the delights and attractions of Pará State, in the Brazilian Amazon, particularly among the riverine populations who have food sovereignty in planting what they consume, and potentially recognized for its health benefits similar to purple açaí.

The results underscore the importance of considering both factors—fruit variety and mesoregion—when studying the physicochemical characteristics of fruits. The significance of interactions indicates that variations in fruit characteristics are not uniform, depending on both the variety and specific mesoregion. This suggests that cultivation strategies and improvements may need to be tailored to different regions and varieties to optimize fruit quality and yield.

## Data Availability

The original contributions presented in the study are included in the article/supplementary material, further inquiries can be directed to the corresponding author.
